# Phenotypic variation and differentiated gene expression of Australian plants in response to declining rainfall

**DOI:** 10.1098/rsos.160637

**Published:** 2016-11-16

**Authors:** Haylee D'Agui, William Fowler, Sim Lin Lim, Neal Enright, Tianhua He

**Affiliations:** 1 Department of Environment and Agriculture, Curtin University, Perth, Western Australia 6845, Australia; 2 School of Veterinary and Life Sciences, Murdoch University, Perth, Western Australia 6150, Australia

**Keywords:** adaptation, drought, climate change

## Abstract

Declining rainfall is projected to have negative impacts on the demographic performance of plant species. Little is known about the adaptive capacity of species to respond to drying climates, and whether adaptation can keep pace with climate change. In fire-prone ecosystems, episodic recruitment of perennial plant species in the first year post-fire imposes a specific selection environment, offering a unique opportunity to quantify the scope for adaptive response to climate change. We examined the growth of seedlings of four fire-killed species under control and drought conditions for seeds from populations established in years following fire receiving average-to-above-average winter rainfall, or well-below-average winter rainfall. We show that offspring of plants that had established under drought had more efficient water uptake, and/or stored more water per unit biomass, or developed denser leaves, and all maintained higher survival in simulated drought than did offspring of plants established in average annual rainfall years. Adaptive phenotypic responses were not consistent across all traits and species, while plants that had established under severe drought or established in years with average-to-above-average rainfall had an overall different physiological response when growing either with or without water constraints. Seedlings descended from plants established under severe drought also had elevated gene expression in key pathways relating to stress response. Our results demonstrate the capacity for rapid adaptation to climate change through phenotypic variation and regulation of gene expression. However, effective and rapid adaptation to climate change may vary among species depending on their capacity to maintain robust populations under multiple stresses.

## 1. Introduction

Bioclimatic modelling of species distributions suggests that extinction rates may increase dramatically in response to future climate change, with potentially large losses in biodiversity [1–5]. These projections raise great concerns about the deleterious consequences globally for biodiverse floras [6]. However, the validity of these extinction predictions is uncertain as critical gaps remain in our knowledge of the intrinsic capacity of species to respond to climate changes through rapid phenotypic and physiological change for better survival (i.e. adaptation). Species may have the potential to mitigate the effects of a changing climate through rapid selection and adaptation that lead to ‘effect dampening’ within a short time frame [7,8].

Mediterranean-type ecosystems (MTEs) are among the most biologically diverse terrestrial ecosystems globally [9,10], and are highly vulnerable to species extinction under global change [11,12]. Both drought and fire play an important role in shaping the structure and composition of MTE vegetation, as the distribution and abundance of plant species is determined primarily by their ability to tolerate water stress and extreme temperatures in the summer, and to re-establish themselves after fire. In Southwestern Australia (SWA), an MTE global biodiversity hotspot, the climate has undergone a dramatic drop in annual rainfall (more than 30%) since the 1970s, with decreases in rainfall most apparent in late autumn and early winter [13,14]. Significant decrease in rainfall is coupled with an increased frequency of extreme drought events [14–16]. Increases in drought are of particular concern because drought has the capacity to cause sudden and extreme vegetation change, especially when combined with fire in such Mediterranean-type shrublands which already have low baseline water levels [15,17].

Plants may respond to climate change by migrating or shifting their geographical range if possible [18]. Recent discoveries have shown that Australian plant species have the capacity to disperse their seeds to distant habitats up to 3 km away in a single dispersal event [19–21]. However, most Australian species seem to have persisted through major climatic changes over the past few million years, rather than moving long distances to track changing climates [22,23]. This supports the idea that plant species may be able to adapt *in situ* to new climatic conditions, to some extent at least, through rapid evolutionary adaptation. Evolutionary adaptation can be rapid [7] and can help species to counter environmental stresses arising from climate change [24]. It is important to understand the capacity of species to tolerate climate change and the mechanisms that might buffer them against the consequences of such changes in environmental conditions.

Plants in SWA offer a unique opportunity to quantify the pace of selection and adaptation to climate change. In fire-prone ecosystems of SWA, many plant species are characterized by cohort regeneration after fire, so that populations are largely single-aged, with stands of fire-killed species being replaced after each fire (for more details see electronic supplementary material, Study system). This means that all individuals in a stand are established in the same year, and have experienced the same environmental circumstances (the same selection filter), while individuals in stands established in other years will have experienced different environmental circumstances, representing different strengths of selection pressure from climate conditions. Fire is patchy every year, and rainfall also varies between years, creating populations established under different strengths of selection pressure. For example, in a year with low rainfall following fire, the populations established in that year will have been filtered by strong selection from drought; conversely, in a wet year following fire, selection pressure from drought would be relaxed. Climate extremes in the year of regeneration may therefore impose a ‘hard’ selection upon those species, and select for stress-tolerant genotypes within a single generation. Many shrub and tree species in SWA are serotinous, with seeds stored in woody fruits in the living canopy for several to many years and released en masse following fire. Individuals subjected to abiotic stress, such as drought, may retain an imprint of this stress that facilitates higher protection from stress in future generations (e.g. [25,26]), and such trans-generational response may be a potential mechanism of rapid adaptation to environmental and climate change. Episodic (cohort) recruitment of perennial plant species in the first year after fire, imposing a specific selection of abiotic stress, offers a unique opportunity to quantify the scope for rapid adaptive response to climate change. Here, we aimed to determine whether seed banks of four fire-killed, serotinous species, *Banksia hookeriana*, *B. leptophylla*, *Hakea costata* and *H. polyanthema*, have the potential to mitigate the effects of a drying climate through rapid expression of drought-tolerant genotypes.

## 2. Material and methods

### 2.1. Glasshouse experiment

Four serotinous species from the family Proteaceae, *Banksia hookeriana* Meisn*.*, *Banksia leptophylla* A.S. George, *Hakea costata* Meisn. and *Hakea polyanthema* Diels., from the biodiverse SWA Kwongan were investigated (for details see the electronic supplementary material). For each species, canopy-stored seeds set 1–2 years prior to the investigation were collected from five sites (three for *Hakea costata*) of different post-fire age at eight locations near Eneabba, Western Australia (electronic supplementary material, table S1). The sites are geographically proximate (2–60 km) and with similar species composition (typical Kwongan vegetation dominated by species from the families Proteaceae and Myrtaceae) and soils (low nutrient, acidic white sands). All sites have experienced the same long-term climate while fire history may vary, as fires are patchy in size and location [27]. Distances between sites are sufficiently large such that genes are not immediately mixed through pollen and seed dispersal after fire [19,20]. All sites were last burnt at least 8 years ago, and so supported mature stands of the selected species. Sites were classified either as average-to-wet winter (HiR; high rainfall populations) or dry winter (LoR; low rainfall populations) based on total rainfall in the first winter/spring following the last fire, with ‘dry’ defined as more than 20% below the long-term average mean winter rainfall at Eneabba (electronic supplementary material, table S2).

Seeds were extracted from woody fruits and germinated at 15°C before being transferred to custom-made pots (15 cm diameter, 100 cm deep—to facilitate the natural pattern of early tap root growth). Seedlings were grown in a temperature-controlled glasshouse (air temperature ranged from 12.9°C to 36.1°C, and soil temperature ranged from 12.4°C to 31.2°C). Seedlings were watered every second day with 200 ml water for four weeks to allow seedlings to establish. Once established, seedlings from HiR and LoR sites for each species were divided into two treatment groups, with up to 60 replicates per species per group and an equal number of seedlings from each location in each group. Seedlings were subjected to either a control (mean winter rainfall at Eneabba over the past 30 years equivalent; approximately 200 ml per plant every second day) or drought regime (equivalent to a 50% decrease in mean winter rainfall at Eneabba; 100 ml per plant every second day) for two weeks and then no water over the next three months simulating severe drought. After three months of growth, half of the seedlings (up to 24, electronic supplementary material, table S4) of each species were harvested for measurement of growth and phenotypic variation in drought resistance traits. The remaining half of the seedlings were grown on without water supply in the greenhouse for a further 12 weeks with mortality recorded each week. For more details, see the electronic supplementary material.

### 2.2. Trait measurements and statistical analysis

After three months, up to 24 plants from each of the treatment groups and sites (HiR and LoR) were harvested for each species. Growth (total dry biomass) and five traits that are related to drought resistance were measured: root length, leaf mass per area (LMA), water content per unit biomass, water content per unit root length and water content per leaf area. Relative fitness for each trait was represented as standardized trait values, with data standardized as (*v *− Min)/(Max* *− Min), where *v* is trait value, and Min and Max are the minimum and maximum values in each trait. We first used canonical discriminant function analysis to summarize drought and growth traits for the overall physiological response of seedlings derived from HiR and LoR sites under average versus droughted growing conditions. We used Wilks's lambda and associated *χ*^2^ statistic as a measure of the difference in overall physiological response between HiR and LoR seedlings of each species. Variation in each growth and drought parameter, and survival between sources of seeds (HiR or LoR) were compared using one-way ANOVAs. Variations between treatments with different sources of seeds (fixed variates), and different watering regimes (covariates) were compared using two-way ANOVAs. In the case of unequal variance, Welch F-tests were used. Median values of trait measurements between seed sources (HiR or LoR) were compared using Kruskal–Wallis tests. Statistical analyses were performed in PAST V3 [28] and SPSS 22 [29]. Significance level was set at *p* < 0.05 for all statistical tests.

### 2.3. Differentially expressed genes in relation to drought treatment

*Banksia hookeriana* was investigated further using transcriptome analyses, due to its known susceptibility to drought [15]. Samples of *B. hookeriana* for genetic analyses were harvested 10 weeks into the drought treatment (as species in SWA generally have three months growth after germination before the onset of the dry season). We collected and pooled five seedlings from each of the two source population groups (HiR and LoR) from both control and drought treatment regimes. Total RNA was extracted and cDNA libraries were constructed and then sequenced using the Illumina HiSeq 2000 Sequencing System (Illumina Inc. San Diego, CA, USA), yielding approximately 50 million 100-bp reads per sample. Sequence data were processed for length and quality, and aligned to protein databases before functional analysis and pathway enrichment analysis (see the electronic supplementary material for more details on pipeline of all data processing steps and parameters). The assembled *B. hookeriana* transcriptome sequences have been deposited in the NCBI database (accession number: GBXB00000000).

## 3. Results

### 3.1. Growth and drought resistance of seedlings under control and drought conditions

We first examined the physiological response in growth and phenotypic traits in simulated drought for the four species. Multivariate analysis through canonical discriminant function suggested significant overall difference in physiological response for six measured growth and drought-related traits between seedlings derived from populations established in years with average-to-above-average winter rainfall (HiR) and those derived from populations established in years with at least 20% below-average winter rainfall (LoR) in *B. hookeriana* and *H*. *costata,* when growing in conditions with full water supply (control). Overall differences in physiological response between HiR and LoR populations were revealed for *B. leptophylla*, *H. costata* and *H. polyanthema* when growing under drought conditions (table 1).

**Table 1.** Canonical discriminant function analysis of overall difference of growth and physiological traits between HiR and LoR seedlings of the four species growing in conditions either with full water supply or with simulated drought. Significant *p*-values in italics (*α* = 0.05).

species	watering regime	Wilks's lambda	probability
*Banksia hookeriana*	full water	0.684	*0.038*
	simulated drought	0.860	0.680
*Banksia leptophylla*	full water	0.850	0.476
	simulated drought	0.690	*0.048*
*Hakea polyanthema*	full water	0.760	0.860
	simulated drought	0.528	*0.049*
*Hakea costata*	full water	0.341	*0.006*
	simulated drought	0.519	*0.024*

**Figure 1.**
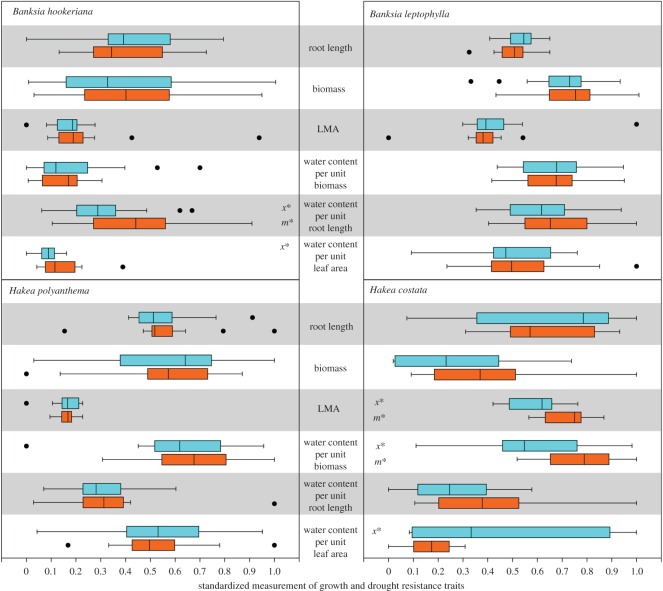
Standardized measurement of growth and drought traits of seedlings derived from HiR (blue) and LoR (orange) populations in the glasshouse experiment with full water supply. Box indicates 25–75th percentile, black dots represent outliers. An ‘*x*’ with an asterisk indicates a significant difference in mean values, and an ‘*m*’ with an asterisk indicates a significant difference in median values between HiR and LoR populations.

When growing in conditions with average water availability, seedlings in two species, derived from LoR populations, *B. hookeriana* and *H. costata*, showed higher relative fitness in four traits than seedlings derived from HiR populations (figure 1). Seedlings of *B. hookeriana* from LoR sites had higher water content per unit root length and higher water content per leaf area than seedlings from HiR sites, indicating higher efficiency in water uptake and water use in those seedlings whose parents were established in drought years compared with those whose parents were established in average-to-wet years. Apart from storing more water per unit biomass, seedlings of *H. costata* from LoR sites developed higher LMA than HiR seedlings (figure 1) when grown in conditions with average water availability.

**Figure 2.**
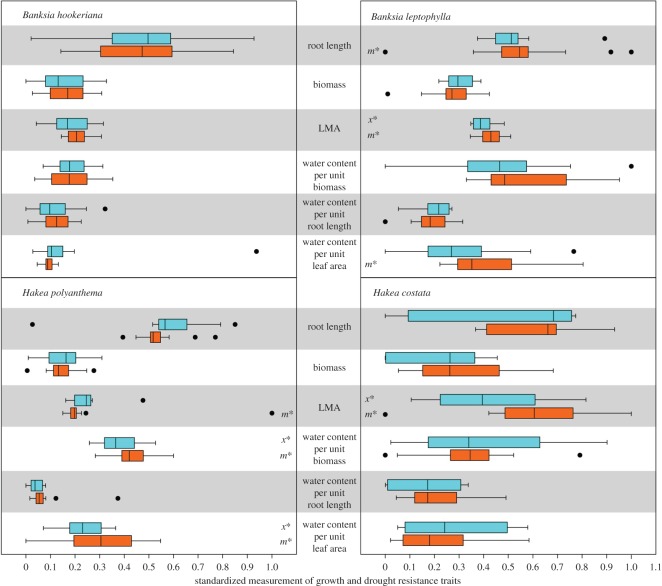
Standardized measurement of growth and physiological traits of seedlings derived from HiR (blue) and LoR (orange) populations in the glasshouse experiment under conditions of water deficit. Box indicates 25–75th percentile, black dots represent outliers. An ‘*x*’ with an asterisk indicates a significant difference in mean values and an ‘*m*’ with an asterisk indicates a significant difference in median values between HiR and LoR populations.

LoR seedlings of *B. leptophylla* and *H. polyanthema* showed no difference in growth and drought resistance traits compared with HiR seedlings when grown in conditions with average water availability (figure 1). When grown under drought conditions, LoR seedlings of *B. leptophylla* had longer roots, higher LMA and higher water content per leaf area than HiR seedlings (figure 2); LoR seedlings of *H. polyanthema* had higher water content per unit biomass and higher water content per unit leaf area than HiR seedlings, and LoR seedlings of *H. costata* had higher LMA than HiR seedlings when grown in drought conditions (figure 2).

Despite the results that improved fitness in relation to drought tolerance was not consistent across all traits and species in relation to their origin, two-way ANOVAs indicated that seedlings from LoR populations generally had higher LMA, higher water content per unit root length, higher water content per unit leaf area, and higher water content per unit biomass than seedlings from HiR populations (table 2). Because climate extremes in the year of regeneration impose a ‘hard’ selection upon those species, such selection would then remove, or decrease the frequency of, less stress-tolerant genotypes. Indeed, LoR populations generally had a smaller proportion of individuals having lower relative fitness in relation to drought resistance than HiR populations (*p* = 0.016, one sample test of *χ*^2^ goodness of fit).

**Table 2.** Two-way ANOVA of each trait with source of seedlings (HiR or LoR) as fixed variants, and different watering regime as covariant. Probabilities (less than 0.10) of overall difference between sources of seedlings are shown. Significant *p-*values in italics (*α* = 0.05).

species	root length	biomass	LMA	water content/ biomass	water content/ root length	water content/ leaf area
*Banksia hookeriana*	—	—	0.073	—	0.095	—
*Banksia leptophylla*	—	—	—	—	0.093	—
*Hakea polyanthema*	*0.040*	—	—	—	*0.045*	—
*Hakea costata*	—	—	*0.015*	*0.045*	—	*0.014*

### 3.2. Mortality under severe drought

**Figure 3.**
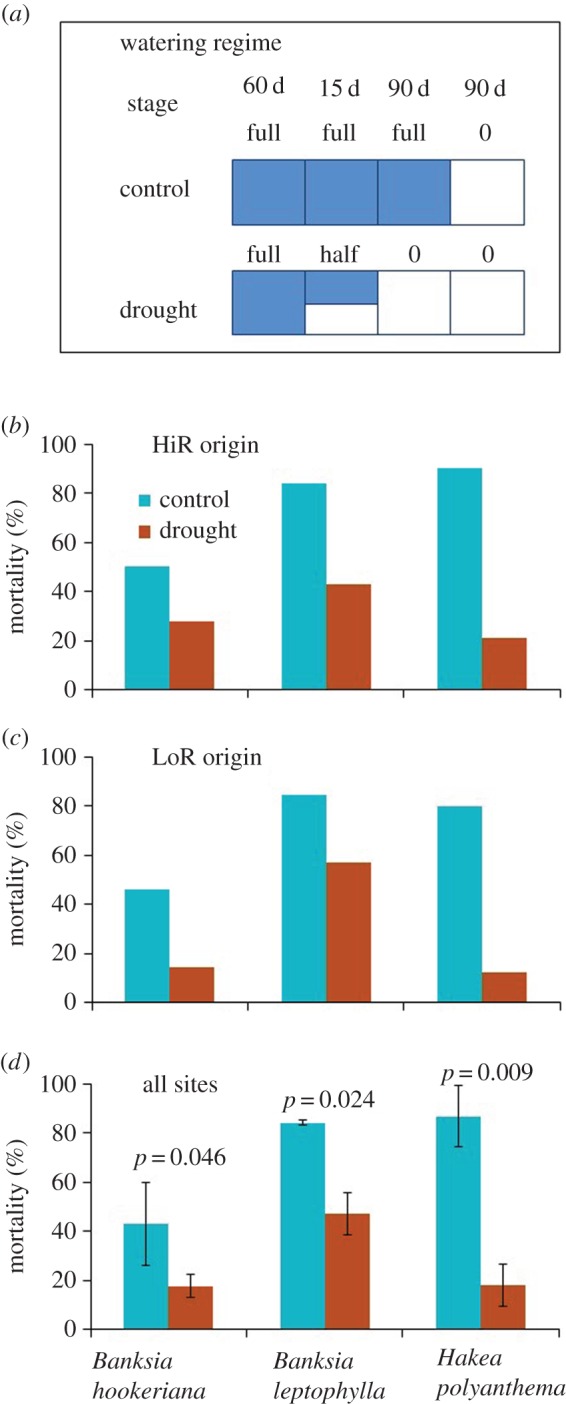
Watering regime and mortality (% at end of experiment) of seedlings derived from HiR and LoR sites of three species in the glasshouse experiment under three to six months of drought (*Hakea costata* was not included due to low survivorship, which left insufficient samples to be monitored further for mortality under drought). (*a*) Watering regime. Four stages were arranged, ranging from 15 days to 90 days over a period of 8.5 months; ‘full’ indicating full water supply (200 ml per plant, every second day) over the stage; ‘half’ indicating half water supply (100 ml per plant, every second day); ‘0’ indicating no water supply. (*b*) Mortality of seedlings from HiR sites; (*c*) mortality of seedlings from LoR sites; (*d*) mortality of seedlings over all sites in the two treatments. The *p*-values are probabilities of equal mortalities of seedlings from HiR and LoR sites (one-way ANOVA).

We further assessed survival under simulated severe drought for three of the four species (there were insufficient *H. costata* seedlings after harvesting for trait measurements) by terminating the water supply after five and a half months and monitoring continued growth and survival for three months (figure 3*a*). At the end of the experiment (i.e. after 8.5 months), seedlings from LoR populations had lower mortality than those from HiR populations (figure 3*b*,*c*). For all three species, seedlings that had been treated with drought at the start of the experiment had significantly lower mortality than seedlings from the control group (figure 3*d*).

### 3.3. Differentiated gene expression and regulation networks in *Banksia hookeriana*

Finally, we assessed differentiated gene expression and regulation networks in seedlings of *B. hookeriana* derived from HiR and LoR populations and grown under simulated drought, versus average water availability (control). Among the 59 064 transcripts assembled from all samples, 8.2% had differentiated expression between samples derived from HiR populations and LoR populations (natural logarithm transformed fold change *t* > 2.5, false discovery rate < 0.05). Gene ontology (GO term) comparison revealed that differentially expressed genes (DEGs) were mostly involved in the oxidation–reduction process, protein phosphorylation, metabolic process, response to stress and regulation of transcripts, regardless of the source of seedlings, when grown in conditions with simulated drought.

Gene regulation networks created through pathway enrichment analysis revealed gene function with significant expression change (*t* > 2.5 or *t* < −2.5) in *B. hookeriana* seedlings derived from HiR and LoR populations and grown in simulated drought conditions when compared with seedlings grown in control conditions. Cellular pathways related to growth, development and metabolism were downregulated in all seedlings, irrespective of the source of populations (table 3), which is consistent with reduced growth observed in seedlings in terms of biomass accumulation when growing in drought conditions. Defence response and response to stress pathways were upregulated in seedlings from both sources when grown in drought conditions; however, HiR and LoR seedlings responded to drought via regulation with different plant hormones. In HiR seedlings, jasmonic acid biosynthesis and metabolism were more active under drought conditions, while in LoR seedlings, salicylic acid and brassinosteroid biosynthesis and metabolism were stronger (table 3). Pathways of programmed cell death were also upregulated in LoR seedlings when under drought conditions.

**Table 3.** Biological pathways with significant expression change in seedlings of *Banksia hookeriana* from HiR and LoR populations growing in drought conditions. Differentially expressed genes were identified by a false discovery rate less than or equal to 0.001 and fold change value greater than 2. Differentially expressed gene clusters were identified by an adjusted *p*-value < 0.05.

seedlings with HiR origin	seedlings with LoR origin
*downregulated pathways*	
developmental growth	cell proliferation
meristem development	meristem development
root morphogenesis	xylem development
stomatal complex morphogenesis	stomatal complex morphogenesis
cell-wall biogenesis	cell-wall biogenesis
peptide transport	
protein phosphorylation	protein phosphorylation
response to gibberellin stimulus	
*upregulated pathways*	
programmed cell death	programmed cell death
jasmonic acid biosynthesis and metabolism	salicylic acid biosynthesis and metabolism
response to salicylic acid stimulus	response to biotic stimulus
response to stress	response to stress
heat acclimation	defence response
	cellular response to nutrient deficiency

### 3.4. Genetic diversity in *Banksia hookeriana*

Intensified selection under droughting may have reduced genetic diversity in *B. hookeriana*. We detected an average of 66 377 SNPs in seedlings derived from LoR populations, with an average of 50.6% heterozygous SNPs, compared with 62.9% heterozygous SNPs in seedlings from HiR populations (with an average of 65 799 SNPs detected). Among the transcripts that belonged to genes in salicylic acid biosynthesis and metabolism, 48.1% SNPs were heterozygous in LoR seedlings, while 62.3% were heterozygous in HiR seedlings.

## 4. Discussion

Both phenotypic plasticity and adaptive evolution may contribute to population persistence in a changing environment [30,31]. Although it is difficult to parse out the relative contributions of adaptive evolution and phenotypic plasticity in our study, we suggest that rapid evolutionary adaptation might be the more significant in our study system. Our LoR and HiR sites were geographically proximate with similar climates and environments (but different fire histories). Significant differentiation in drought-related traits of seedlings from LoR and HiR sites was revealed in a common garden experiment using average water supply versus water-deficient growing conditions. Moreover, our transcriptome analysis of *B. hookeriana* revealed that differentiated phenotype was related to differentiated expression of genes, indicating that the adaptive mechanism is heritable through natural selection or epigenetic processes (e.g. [32]). Climate, acting as an environmental filter (i.e. rainfall change) at the time of population establishment may select for drought-resistant alleles [24], resulting in a more drought-tolerant population compared with its parent population. On the other hand, phenotypic plasticity may also determine the immediate response of natural populations to changing climate [33], because phenotypic plasticity may be adaptive and can evolve rapidly in response to selection if it has a heritable genetic basis, for example, through epigenetic processes such as DNA methylation in generating plasticity [32,34].

Our glasshouse experiment revealed that climate changes (declining rainfall in this case) can drive adaptive morphological change in a single generation. Despite such morphological changes not being consistent across all studied species, our results provide evidence of general presence of positive adaptation to drought. Plants with higher LMA are more water-use efficient in terms of assimilation to transpiration rate [35], and in our glasshouse experiment, seedlings of *B. leptophylla* and *H. costata* from populations that had been filtered by drought (i.e. LoR) developed higher LMA when growing under water deficit conditions but not when growing under control conditions. In water-limited habitats, such as those here, the rapid elongation of the root increases the chances of first year seedlings maintaining contact with receding soil water over the first summer [36,37], which is the key to successful seedling recruitment following fire. Seedlings of *B. leptophylla* from populations that experienced the drought filter grew deeper roots than seedlings from populations that did not experience the drought filter. Seedlings from populations that had been filtered by drought might also be more efficient in water uptake, as measured by water content per root length in *B. hookeriana*, and may have more water for transpiration per unit leaf area.

Water deficit led to significant changes in gene expression in seedlings derived from both HiR and LoR populations of *B. hookeriana*, with seedlings actively reprogramming their metabolism, growth and response to stress. In response to water deficit, cellular pathways of stress avoidance and tolerance were upregulated to promote survival; pathways related to growth were generally downregulated. Therefore, plants were able to redirect resources from growth to stress-resistance functions for increased chance of survival [38]. The most significant results from our transcriptome analysis are that LoR-derived seedlings upregulated cellular pathways of salicylic acid biosynthesis and metabolism (as distinct from jasmonic acid biosynthesis and metabolism in HiR-derived seedlings), and programmed cell death. Salicylic acid is involved in a range of cell activities as a response to stress, enhancing tolerance to heat, cold, and drought stress, regulating cell growth, regulating stomatal movement and photosynthetic activity in guard cells of stomata, and initiating flowering and reproduction under stress conditions and cell starvation [39,40]. Salicylic acid has antagonistic effects on jasmonic acid signalling downstream [41], and modifies transcriptional regulators that are involved in suppression of jasmonic acid-dependent genes [42], suggesting a deep layer mechanism of stress adaptation of activating salicylic acid biosynthesis and metabolism.

Programmed cell death is thought to be a mechanism of adaptive response to stress, maintaining cell survival under stress conditions by allowing the degradation and recycling of non-essential components of the cell [43,44]. The adaptive advantages of activating these two cellular pathways (i.e. degradation and recycling of non-essential components) are apparent, as we recorded much lower mortality under drought than control conditions in our glasshouse experiment. The ability to activate these two biological processes after a selection filter in a single generation suggests that there may be an intrinsic capacity for rapid adaptation to stress, probably from standing genetic variation within the population, rather than from new mutations [45], or through an epigenetic process such as DNA methylation resulting in phenotypic plasticity [33].

Directional selection resulting in phenotypic change may increase the fitness of an organism; it also could decrease genetic variability in adaptive evolution [7]. Our results suggest that drought may have selected for homozygotes associated with salicylic acid biosynthesis and metabolism, and reduced genome-wide heterozygosity. It is likely that the homozygous state of those drought-related genes could contribute to upregulated expression, and therefore confer higher fitness when under stress conditions. Consistent with the observed lower mortality rate in the glasshouse under simulated drought, the lower mortality of natural *B. hookeriana* populations with lower microsatellite DNA heterozygosity was also observed after the severe 2006 drought in SWA [15]. The temporal and spatial heterogeneity of selection suggested by this study, together with high gene flow via pollen [46] and seed dispersal [19] might lead to a reservoir of adaptive genetic variation in *B. hookeriana* and other co-occurring species that facilitates rapid adaptation to a changing climate. We observed a considerable number of genes with significant expression change when under drought, suggesting that the genetic basis of adaptation to a drier climate is strongly multigenic.

Our results suggest that some species and ecosystems might be more resilient to climate change than we currently believe, with adaptive evolution through natural selection and/or heritable phenotypic plasticity as results of epigenetic processes within a relatively short time frame [8], in our case, a single generation. Plant communities in biodiverse SWA may be able to tolerate further changes in rainfall through rapid adaptive evolution. Our results suggest drought experienced by a population results in reduced growth, but natural selection across this fitness differential results in a population that is better adapted to water deficit conditions, which represents potential for adapting to a drying climate. For this process to occur, two conditions must be met. First, there must be genetic variation within the population that allows a physiologically beneficial response to low water availability. A study on adaptive genetic variation in response to rainfall and temperature in *B. attenuata* (usually co-occurring with species studied here) indicated that even populations occurring in wet habitats have genetic variation favouring survival under dry conditions [47]. However, directional selection could deplete population genetic variation, which limits the species' adaptive potential for other stresses that require different suites of genes and regulation networks. Indeed, we observed that LoR populations had fewer individuals with lower fitness specific to drought resistance than HiR populations, indicating lower genetic variation within LoR populations than HiR populations. Second, populations must be robust, with high reproductive capacity. This second condition is important because the capacity to respond favourably to stress requires a balance of growth and survival [48,49]. Populations experiencing selection may pay a selective cost in terms of reduced growth, as we observed in our glasshouse experiment, which could lead to lowered reproductive potential [50,51]. Populations impacted by climate change, or by other stresses, such as frequent fire in SWA [6], may have low population growth and reduced capacity to cope with selective impacts of a drying climate. Our glasshouse experiment revealed poorer early growth of seedlings from populations that experienced drought selection than seedlings from populations that did not experience drought selection, even under favourable growing conditions. In conclusion, although plant species in SWA may possess the capacity for rapid adaptation to a drying climate, the extent of rapid adaptation is finite and maintenance of robust populations in the future is an important part of any climate response strategy. Future studies are needed to empirically test the effect of loss of genetic diversity through rapid evolution in response to a drying climate, or other stressors, on overall population variability.

## Supplementary Material

Supplementary Materials and Methods

## Data accessibility

Electronic supplementary material is available on the Journal's website. The assembled *B. hookeriana* transcriptome sequences have been deposited in the NCBI database (accession number: GBXB00000000). Correspondence and requests for materials should be addressed to H.D.
