# Supplementary material for: Phenotypic variation and differentiated gene expression of Australian plants in response to declining rainfall
Source: R Soc Open Sci. 2016 Nov 16;3(11):160637. doi: 10.1098/rsos.160637 (PMC5180152; doi:10.1098/rsos.160637)
Supplement: Supplementary Materials and Methods [file rsos160637supp1.docx]

# Supplementary Materials

## Study System

Mediterreanean-type ecosystem kwongan vegetation of the Eneabba sandplains was selected as the study system. It is located within the South West Australian Floristic Region, an internationally recognised biodiversity hotspot with at least 7,380 native plant taxa, and a high level of endemism (*1*). *Banksia* woodland is the dominant form of vegetation in the area, with taxa from the families Proteaceae and Myrtaceae particularly prominent. Species from the family Proteaceae were chosen for the study. Proteaceae is a dominant family within the Mediterranean climate areas of Southwestern Australia (*1*), and species within this family display various adaptations to the infertile soils, hot temperatures, low rainfall and recurrent fires to which they are exposed (*2*). These adaptations include canopy stored seed banks, with seeds protected in woody fruits which crack open with the heat of fire, resulting in seed release on to a nutrient enriched soil surface where they germinate with the onset of winter rains.

The study area, on the Eneabba sandplain, 275 - 300 km north of Perth, Western Australia, has a Mediterranean-type climate characterised by cool wet winters and hot dry summers (*3*). Mean annual rainfall at Eneabba is 491 mm, with 80% of rainfall occurring in the winter months between May and September (Eneabba Weather Station number 008225; *4*). Mean maximum temperature is 27.9˚C, and mean minimum temperature is 13.6 ˚C while the mean maximum temperature of the hottest month (February) is 36.4˚C (*5*). Winter rainfall has decreased in the region since the 1970s (Fig. S1). The study system possesses low baseline water availability, and plants rely on winter rainfall to survive the dry summer and autumn months. As such, further decrease in rainfall is of particular concern, as drought has the capacity to cause sudden and extreme vegetation change (6*, 7*).

**Figure S1:** Total annual rainfall at Eneabbba since 1964, with rain falling in winter (June-August; shaded bars) also indicated (*4*).

## Study species, population selection and seed collection

Four species characterised by canopy seed storage and cohort recruitment after fire were selected; *Banksia hookeriana* Meisn*.*, *Banksia leptophylla* A.S. George, *Hakea costata* Meisn., and *Hakea polyanthema* Diels, all belonging to the family Proteaceae*. Banksia hookeriana* was chosen for further genetic studies due to its greater susceptibility to drought (*6*).

Populations of the four species were sampled from stands with different post-fire ages at eight locations near Eneabba, Western Australia (Table S1). Seed source locations used were classified as high rainfall (HiR) if they received an average to above-average rainfall in the first winter after fire, or low rainfall (LoR) if they experienced drought (less than 80% of average rainfall) in the first winter following fire (Table S2; *4*). The time elapsed since last fire for each site was provided by the Department of Parks and Wildlife (Western Australia), the government agency responsible for managing and monitoring fire across the state, and was also verified by the age of plants at each site using annual stem node counts for *Banksia hookeriana* (following *8*).

Each of the four species were sampled from five of eight locations (except for *H. costata*, which was only present at three locations), from June-August 2013, with fruits harvested from 15 individuals per species, with the aim of collecting 150 viable seeds per species per site. The seeds collected were set over the two years prior to collection to maximise seed viability and to minimise environmental variation in any factors affecting seed set across years. The seed collection site locations and details are shown in Table S1.

Table S1: Site categorisation, location and species collected. LoR (Low Rainfall) refers to low rainfall years and HiR (High Rainfall) to average/above average rainfall years.

| Site | Year last burned/plants established | Winter/spring rainfall (mm) and category | GPS coordinates | Species collected | | | |
| --- | --- | --- | --- | --- | --- | --- | --- |
|  |  |  |  | *Banksia hookeriana* | *Banksia leptophylla* | *Hakea polyanthema* | *Hakea costata* |
| 1 | 2006 | 260 (LoR) | S 29°38’32”  E 115°12’43” |  | ✔ | ✔ |  |
| 2 | 2002 | 320 (LoR) | S 29°37’03”  E 115°12’00” |  | ✔ | ✔ | ✔ |
| 3 | 2000 | 311 (LoR) | S29°45’26”  E115°11’11” | ✔ |  | ✔ | ✔ |
| 4 | 2002 | 320 (LoR) | S 29°52’15”  E 115°14’54” | ✔ | ✔ |  |  |
| 5 | 2005 | 429 (HiR) | S 29°56’34”  E 115°16’00” | ✔ |  |  |  |
| 6 | 2005 | 429 (HiR) | S 29°45’27”  E 115°11’14” | ✔ |  |  |  |
| 7 | 1998 | 401 (HiR) | S 29°35’50”  E 115°10’12” |  | ✔ | ✔ | ✔ |
| 8 | 1991 | 512 (HiR) | S 29°37’03”  E 115°12’02” | ✔ | ✔ | ✔ |  |

Table S2: Criteria for classifying rainfall at Eneabba. Long term average was calculated using data from 1965 to 2012.

|  | Annual rainfall (mm) | Winter/spring rainfall (mm) |
| --- | --- | --- |
| Long term average (Average rainfall) | 492 | 426 |
| Long term average -20% (Low rainfall-LoR) | 394 | 341 |

## Seed preparation and germination

Seed was extracted from fruits of *Hakea* species by allowing fruits to air dry and open naturally. Seed was extracted from fruits of *Banksia* species by first scorching cones with a gas torch until follicles split open, then soaking cones in cold water over night, and drying cones in an oven at 60°C until follicles were open wide enough for seed to be extracted with a pair of forceps.

Seed was surface sterilised in a solution of 20% sodium hypochlorite with a drop of Tween 80 surfactant (polyoxyethylene sorbitan mono-oleate) then placed under pressure in a vacuum chamber for five minutes, removed from the vacuum for five minutes, and returned to the vacuum chamber for another five minutes, then rinsed three times in sterile deionised water (*9*). Sterilised seed was placed into Petri dishes of varying size (120 mm for *H. costata* seed, and 150 mm for large *H. polyanthema*, *B. hookeriana* and *B. leptophylla* seeds) containing two sheets of Whatman No. 1 filter paper, 2 cm^2^ pieces of Wettex sponge (4 pieces for 120 mm Petri dishes, and 5 pieces for 150 mm Petri dishes), and water (13 mL for 120 mm Petri dishes, 16 mL for 150 mm Petri dishes). Petri dishes were then placed in a germination cupboard at 15°C for one month, until all viable seeds had germinated.

## Greenhouse experiment

Germinants were removed from the germination cupboard and sown into 100 cm x 15 cm diameter PVC tube pots containing low nutrient, acid sand (Bassendean sand; *10*) similar in type to sands at Eneabba, with three germinants of the same species from the same site sown per tube, and thirty replicates made of each. After a month of growth, ash (produced by burning local native plant species) was added to the surface of the soil at a rate of 6.68 grams (one tablespoon) per tube or 0.03789 grams of ash per cm^2^ (*11*). Seedlings were grown in a greenhouse at Murdoch University, Perth, Western Australia, where they were watered every second day with 200 mL of water (simulating Eneabba mean winter rainfall equivalent) for one month to allow seedlings to establish, after which seedlings were harvested down to one seedling per tube. Once established, seedlings were divided into two treatment groups (15 replicates per treatment) and subjected to either a control (mean winter rainfall at Eneabba over the past 30 years equivalent; 200 mL per plant every second day) or drought regime (a 50% decrease in mean winter rainfall at Eneabba; 100 mL per plant every second day) over a period of three months (Table S3).

Table S3: Watering regime in simulated drought and control treatments. 100% water refers to 200 mL of water per plant, every second day, 50% water refers to 100 mL of water per plant every second day, and 0% water refers to no water.

| Treatment | Time | | | |
| --- | --- | --- | --- | --- |
|  | 2 months | 2 weeks | 3 months | 3 months |
| Control | 100% water | 100% water | 100% water | 0% water |
| Drought | 100% water | 50% water | 0% water | 0% water |

Samples of *Banksia hookeriana* to be used for genetic analyses were harvested after two months (see below). After three months up to 24 plants from each of the treatment groups and sites were harvested for each species (Table S4), and measurements were taken of the number of leaves, leaf area (of 5 leaves per plant, using the freeware program ImageJ; *12*), leaf thickness and total fresh biomass. Seedlings were dried in an oven at 80˚C after which total dry biomass and biomass of individual leaves (5 per plant) were recorded. Leaf mass per area was calculated as leaf dry weight/leaf area. Water content was obtained as total fresh biomass – total dry biomass. Remaining seedlings were kept growing in the greenhouse for 12 weeks, with mortality recorded each week.

**Table S4:** Allocation of seedlings to experiments. Number of seedlings used for physical measurements and mortality data are listed. Eight seedlings were used from each site and data for seedlings from sites of the same type were pooled for analysis.

| Species | Treatment | Type | Sites | Seedlings used for measurements | Seedlings used in mortality experiment |
| --- | --- | --- | --- | --- | --- |
| *Banksia hookeriana* | 100% | Wet | 3 | 24 | 16 |
|  |  | Dry | 2 | 16 | 24 |
|  | 50% | Wet | 3 | 24 | 18 |
|  |  | Dry | 2 | 16 | 14 |
| *Banksia leptophylla* | 100% | Wet | 2 | 16 | 13 |
|  |  | Dry | 3 | 24 | 19 |
|  | 50% | Wet | 2 | 16 | 14 |
|  |  | Dry | 3 | 24 | 21 |
| *Hakea polyanthema* | 100% | Wet | 2 | 16 | 10 |
|  |  | Dry | 3 | 24 | 20 |
|  | 50% | Wet | 2 | 16 | 14 |
|  |  | Dry | 3 | 24 | 16 |
| *Hakea costata* | 100% | Wet | 1 | 7 | 0 |
|  |  | Dry | 2 | 16 | 4 |
|  | 50% | Wet | 1 | 8 | 0 |
|  |  | Dry | 2 | 16 | 5 |

**RNA sequencing and differential expression of genes**

## *Experimental material*

Differential gene expression analyses were performed using seedlings of *B. hookeriana*, as it is the most drought-susceptible of the four study species (*6*). Leaf material was collected after two months of growth, with five seedlings harvested from each of the four treatment groups of seedlings: 1) those descended from plants recruited in a HiR year treated with 50% mean annual winter rainfall equivalent; 2) those descended from plants recruited in a HiR year treated with 100% mean annual winter rainfall equivalent; 3) those descended from plants recruited in a LoR year, treated with 50% mean annual winter rainfall equivalent; and 4) those descended from plants recruited in a LoR year, treated with 100% mean annual winter rainfall equivalent. Leaf material was cleaned with DEPC water (Diethylpyrocarbonate water, an RNase enzyme inhibitor), and stored in RNAlater (Life Technologies Australia Pty Ltd.).

***RNA Sequencing***

For each treatment, leaf samples were collected from four seedlings and pooled. Samples were stored in RNAlater RNA Stabilization Reagent (Thermo Fisher Scientific Inc. Perth) before being processed. RNA extraction, cDNA library preparation, and sequencing was carried out by Beijing Genomics Institute BGI (Shenzhen, China). Briefly, samples were homogenised, total RNA was isolated from leaf samples using Total RNA Purification System (Invitrogen™), RNA quality and concentration were checked with Nanodrop™ and Agilent 2100 Bioanaylzer. Poly (A) mRNAs were isolated using beads with oligo(dT) (Qiagen GmbH, Hilden, Germany). The mRNA was fragmented and used as a template. cDNAs were obtained using a random hexamer to synthesize first-strand. The short cDNA fragments were then connected using sequencing adapters. The paired-end library (100 base pair insertion) was prepared following the protocol of the Illumina TruSeq RNA Sample Preparation Kit (Illumina), and the library was sequenced using Illumina HiSeq^TM^ 2000 (Illumina Inc., San Diego, CA, USA). Raw reads were quality controlled by removing adaptor sequences, empty reads and low quality reads.

***Transcriptome De Novo Assembly***

Transcriptome *de novo* assembly was carried out using a de Bruijn graph and the short read assembling program Trinity that consists of 3 independent programs; Inchworm, Chrysalis, and Butterfly (*13, 14*). Inchworm first assembled the RNA-seq data into the unique sequences of transcripts (contigs) with a certain overlap length (k-mer = 25) and minimum overlap coverage of 3 reads. The resulting contigs were then clustered by Chrysalis into clusters and complete de Bruijn graphs were constructed for each cluster. In the final step, Butterfly processed the individual graphs in parallel, tracing the paths that reads and pairs of reads take within the graph, ultimately reporting full- length transcripts for alternatively spliced isoforms (unigenes).

***Transcriptome De Novo assembly of sequence read of Banksia hookeriana***

An average of 47,287,067 clean reads with total average of 4,728,706,650 nt and an average GC content of 45.73% were generated from four samples of *Banksia hookeriana*. We defined the reads with Q > 20 and no ambiguous sequences (N) as high-quality reads, which resulted in 46,289,310 (97.89%) clean reads. An average of 99,439 contigs were assembled from those clean reads. The length of contigs ranged from 100 nt to 12,556 nt with an average length of 402 nt. Trinity’s Butterfly software was used to connect the contigs to form a unigene. This method assembled 59,063 unigenes, where 25, 912 unigenes were distinct clusters, and 33, 151 unigenes were distinct singletons. The mean size of unigenes was 1098 bp with lengths ranging from 300 bp to > 3,000 bp, and an N50 of 1813 nt. The assembled sequences have been deposited in the NCBI database (accession number: GBXB00000000).

***Differential Expression of Genes and Pathway Enrichment Analysis***

A rigorous algorithm has been developed to identify differentially expressed genes between two samples by BGI based on ‘The significance of digital gene expression profiles’ (*15*). We used a very stringent cutoff, “FDR” (False Discovery Rate) of ≤0.001 and fold change value of 2 for identifying differentially expressed genes. We further studied the biological significance of differentially expressed genes by an enrichment analysis of gene ontology (GO) terms using Blast2GO (version 2.3.5; <http://www.blast2go.org/>). GO functional enrichment analysis was carried out, and KEGG pathway analyses were performed using Path_finder software against the KEGG databases (*16*). Statistically significant over-representation of GO categories in response to the drought treatment was determined. A list of differentially expressed genes clusters was reported after correcting for multiple comparisons (adjusted p-value < 0.05).

## References

1. S.D. Hopper, P. Gioia, The Southwest Australian Floristic Region: Evolution and conservation of global hot spot of biodiversity. *Annual Review of Ecology, Evolution, and Systematics* **35,** 623-650 (2004).
2. P.Groom, B.B. Lamont, Plant life of Southwestern Australia: adaptations for survival, De Gruyter Open (2015).
3. A.F.W. Schimper, W.R. Fisher, P. Groom, I.B. Balfour, Plant-geography upon a physiological basis. 507. (Clarendon Press, 1903).
4. Bureau of Meteorology. Monthly Rainfall at Eneabba Weather Station (2015a). Available from: www.bom.gov.au.
5. Bureau of Meteorology .Monthly Mean Maximum and Minimum Temperature at Eneabba Weather Station (2015b). Available from: www.bom.gov.au.
6. T. He, B.B. Lamont, High microsatellite genetic diversity failed to predict greater population resistance capacity in response to extreme climate change. *Conserv. Genet.* **11,** 1445-1451 (2010).
7. C.J. Yates, A. McNeill, J. Elith, G.F. Midgley, Assessing the impacts of climate change and land transformation on *Banksia* in the South West Australian Floristic Region. *Divers. Distrib.* **16,** 187–201 (2010).
8. B.B. Lamont, *Fire responses of sclerophyll shrublands- A population ecology approach with particular reference to the genus Banksia*. In: J.R. Ford (editor), Fire ecology and management in Western Australian ecosystems, Perth: Curtin University, pages 41-46 (1985).
9. K. Downes. B.B. Lamont, M. Light, J. van Staden, The fire ephemeral *Tersonia cyathiflora* (Gyrostemonaceae) germinates in response to smoke but not the butenolide 3-methyl-2H—furo[2,3-c]pyran-2-one. *Ann. Bot.* **106,** 381-384 (2010).
10. W.M. McArthur, E. Bettenay, Development and distribution of soils of the Swan Coastal Plain, Western Australia. CSIRO Australian Soil Publication Number 16 (1960).
11. N.J. Enright, D. Goldblum, P. Ata, D.H. Ashton, The independent effects of heat, smoke and ash on emergence of seedlings from the soil seed bank of a heathy *Eucalyptus* woodland in Grampians (Gariwerd) National Park, western Victoria. *Austr. J. Ecol.* **22,** 81-88 (1997).
12. W.S. Rasband, ImageJ, U.S. National Institutes of Health, Bethesda, Maryland, USA. Available from: http://imagej.nih.gov/ij/, 1997-2014.
13. M.G. Grabherr, B.J. Haas, M. Yassour, J.Z. Levin, D.A. Thompson, I. Amit, X. Adiconis, L. Fan, R. Raychowdhury, Q. Zeng, Z. Chen, E. Mauceli, N. Hacohen, A. Gnirke, N. Rhind, F. di Palma, B.W. Birren, C. Nusbaum, K. Lindblad-Toh, N. Friedman, A. Regev, Full-length transcriptome assembly from RNA-Seq data without a reference genome. *Nature Biotechnology* **29,** 644-652 (2011).
14. B.J. Haas, A. Papanicolaou, M. Yassour, M. Grabherr, P.D. Blood, J. Bowden, M.B. Cougar, D. Eccles, B. Li, M. Lieber, M.D. Macmanes, M. Otto, J. Orvis, N. Pochet, F. Strozzi, N. Weeks, R. Westerman, T. William, C.N. Dewey, R. Henschel, R.D. Leduc, N. Friedman, A. Regev, De novo transcript sequence reconstruction from RNA-seq using the Trinity platform for reference generation and analysis. *Nature Protocols* **8,** 1494-1512 (2013).
15. S. Audic, J.M. Claverie, The significance of digital gene expression profiles. *Genome Research* **7,** 986–995 (1997).
16. M. Kanehisa, M. Araki, S. Goto, M. Hattori, M. Hirakawa, M. Itoh, T. Katayama, S. Kawashima, S. Okuda, T. Tokimatsu, Y. Yamanishi, KEGG for linking genomes to life and the environment. *Nucleic Acids Research* **36,** D480-D4 (2008).
